# Krüppel-like factor 8 regulates VEGFA expression and angiogenesis in hepatocellular carcinoma

**DOI:** 10.1038/s41598-018-35786-6

**Published:** 2018-11-27

**Authors:** Sanuo Cheng, Xingping Zhang, Yali Xu, Xiaobo Dai, Jiachu Li, Tao Zhang, Xiaopin Chen

**Affiliations:** 1 Department of Oncology, The First Affiliated Hospital of Chongqing Medical University, Chongqing, China; 2 Clinical Medical College, Chongqing Medical and Pharmaceutical College, Chongqing, China; 3 Department of Geriatrics, Chongqing General Hospital, Chongqing, China

## Abstract

Tumor angiogenesis plays a critical role in hepatocellular carcinoma (HCC) development and progression, but its mechanism is unclear. Krüppel-like factor 8 (KLF8) is a transcription factor that plays an important role in HCC progression. Here, we investigated the role of KLF8 in angiogenesis in HCC and its possible mechanism. Immunohistochemistry, quantitative RT-PCR, western blotting, promoter reporter assays, chromatin immunoprecipitation (ChIP), and chicken chorioallantoic membrane (CAM) and nude mouse tumor models were used to show that the mRNA and protein expression levels of KLF8 and VEGFA are highly correlated in HCC tissue samples. The up-regulation of KLF8 increased VEGFA protein levels and induced VEGFA promoter activity by binding to the CACCC region of the VEGFA promoter. In addition, KLF8 regulated HIF-1α and Focal adhesion kinase (FAK) expression. The PI3K/AKT inhibitor LY294002 inhibited KLF8-induced VEGFA expression, whereas PI3K/AKT signaling pathway proteins, such as P-PDK1(Ser241) and P-AKT(Thr308), were decreased significantly. KLF8-overexpressing HCC cells had a higher potential for inducing angiogenesis. Thus, our results indicate that KLF8 may induce angiogenesis in HCC by binding to the CACCC region of the VEGFA promoter to induce VEGFA promoter activity and through FAK to activate PI3K/AKT signaling to regulate HIF-1α expression levels.

## Introduction

Hepatocellular carcinoma (HCC) is the third most common cause of cancer-related death. HCC is a hypervascular tumor characterized by neovascularization, and tumor angiogenesis plays a critical role in its development and progression^1^. Currently, HCC lacks effective systemic therapies, but antiangiogenic therapy is promising for treating HCC.

Vascular endothelial growth factor (VEGF) is the most important angiogenic factor in HCC. VEGF is more highly expressed in human HCC specimens than in non-tumorous liver specimens^2^. There is a strong association between VEGF immunostaining and angiographic vascularity^3^, and VEGF over-expression in tumor cells is directly correlated with tumor angiogenesis in HCC^4^. VEGF expression may be up-regulated through the hypoxia-inducible factor 1-alpha (HIF1-a) pathway^5,6^. The hepatitis B virus (HBV)-encoded transcriptional activator HBV-X protein (HBx) can activate VEGF through the HIF1-a pathway by enhancing the transcriptional activity of HIF-1α^7,8^. The mechanism of VEGF up-regulation in HCC, however, remains unclear.

KLF8 is a member of the Krüppel-like C2H2 zinc-finger transcription factor family of proteins^9^, KLF8 induces tumor cell epithelial-to-mesenchymal transition (EMT), maintains the invasive potential of cancer, and plays a crucial role in the metastatic progression of human carcinoma^10–12^. In our previous research, we found that KLF8 was overexpressed in highly metastatic HCC cell lines and in samples from patients with recurrent HCC. KLF8 up-regulation promoted HCC cell proliferation and invasion and inhibited apoptosis; the over-expression of KLF8 increased HCC progression and metastasis^13^.

In this study, we found that the expression levels of KLF8 and VEGFA were highly correlated in HCC tissue samples, and KLF8 up-regulation induced VEGFA expression in HCC cell lines. KLF8 up-regulation also induced VEGFA promoter activity, and KLF8 bound to the CACCC region of the VEGFA promoter. Furthermore, KLF8 regulated HIF-1α expression in HCC cells. A CAM model and a nude mouse tumor model indicated that KLF8 up-regulation in HCC cells had a higher potential for inducing angiogenesis. KLF8 might activate the PI3K/AKT signal pathway through FAK to increase P-PDK1(Ser241) levels and consequently P-AKT(Thr308) or P-AKT(Ser473) and HIF-1α levels to induce VEGFA protein expression.

## Results

### KLF8 expression and VEGFA expression are highly correlated in HCC tissues at the mRNA and protein levels

Total RNA was prepared from 50 fresh HCC tissue samples using Trizol (Invitrogen), and complementary DNA (cDNA) was then synthesized using the Superscript First-Strand Synthesis System. The mRNA levels of KLF8 and VEGFA in the same liver cancer samples were detected by qPCR, and the relative Ct values for KLF8 and VEGFA were 8.29 ± 2.01 and 4.63 ± 1.32, respectively.

**Figure 1:**
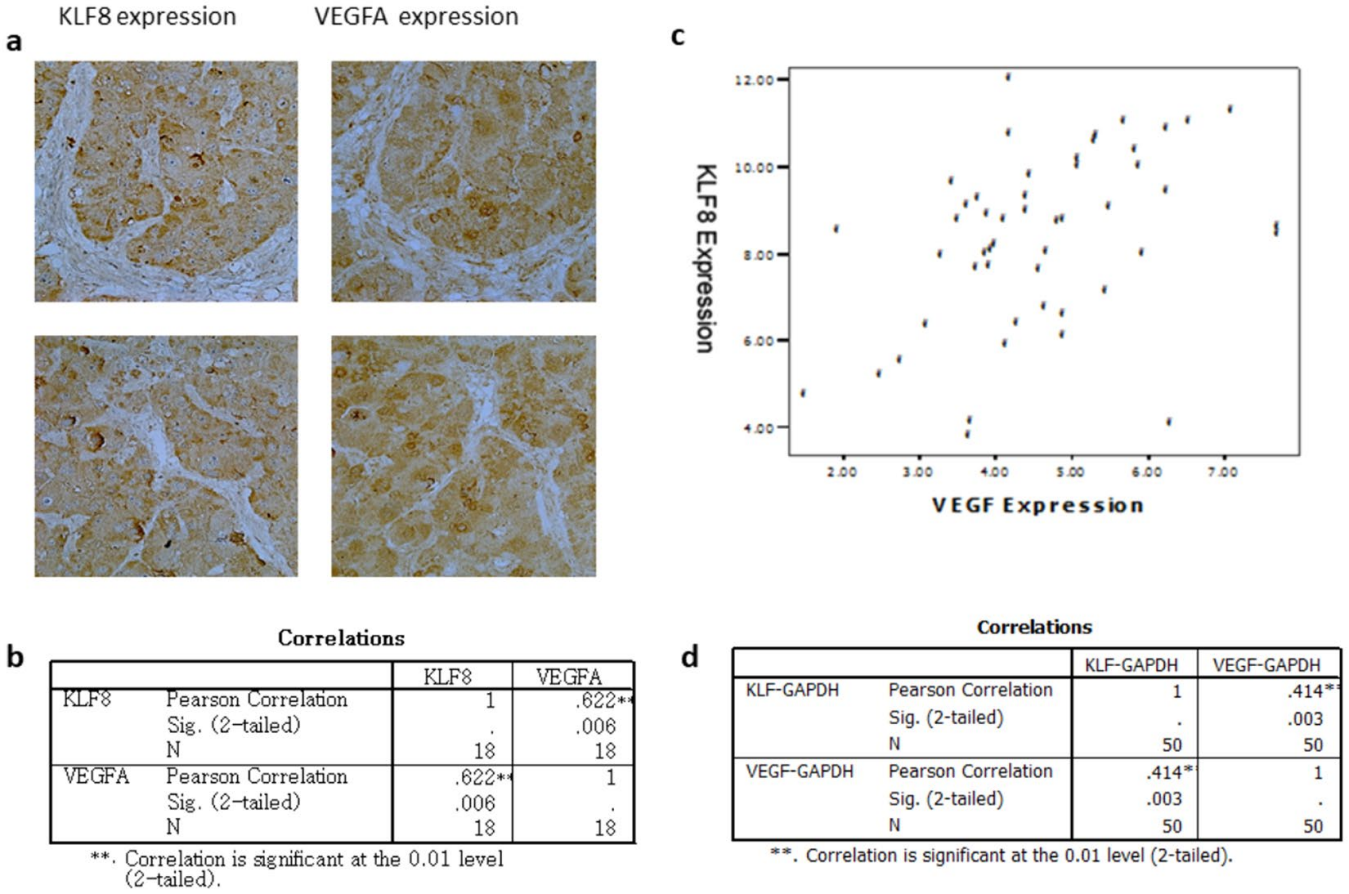
The mRNA and protein levels of KLF8 and VEGFA are highly correlated in HCC. (**a**) KLF8 and VEGFA protein expression levels were detected in paraffin-embedded HCC specimens by immunohistochemistry staining. Integrated densities were measured by ImageJ. (**b**) Pearson’s correlation analysis indicated that KLF8 protein expression was highly correlated with VEGFA protein expression, r = 0.622, p < 0.05. (**c**) mRNA expression levels of KLF8 and VEGFA in 50 fresh HCC tissue samples were detected by qRT-PCR, and the relative expression levels were calculated using the 2^−ΔΔCt^ method. (**d**) Pearson’s correlation analysis indicated that KLF8 mRNA expression was highly correlated with VEGFA mRNA expression, r = 0.414, p < 0.05.

VEGFA and KLF8 protein expression levels in 18 HCC samples were detected by immunohistochemistry staining, and the integrated staining densities were measured by ImageJ. The integrated staining density was 111.91 ± 37.97 for KLF8 and 113.98 ± 53.07 for VEGFA. Pearson’s correlation analyses indicated that KLF8 and VEGFA in the HCC samples were highly correlated at the protein level (r = 0.62, *p* < 0.01) (Fig. 1a,b) and the mRNA level (r = 0.41, *p* < 0.01) (Fig. 1c,d).

### KLF8 up-regulation increases VEGFA mRNA and protein levels in HCC

**Figure 2:**
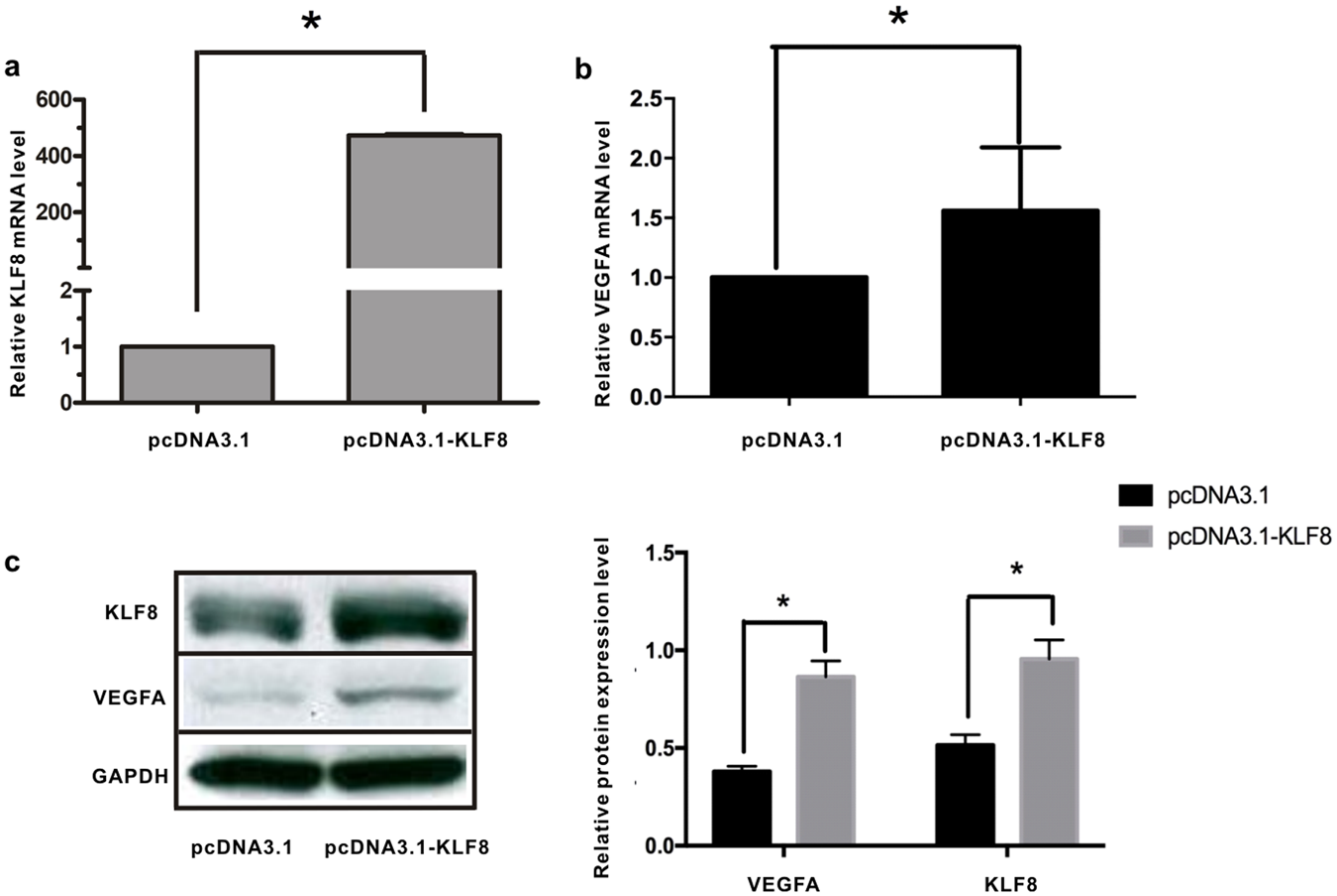
The mRNA and protein levels of VEGFA are increased in KLF8-overexpressing HCC cell lines. (**a**) KLF8 mRNA levels were significantly higher in pcDNA3.1-KLF8-transfected SMMC7721 cells than in pcDNA3.1-transfected SMMC7721 cells, the relative KLF8 mRNA level was 472.66 ± 8.65 (*P* < 0.001, n = 3). (**b**) VEGFA mRNA expression levels were increased significantly in pcDNA3.1-KLF8-transfected SMMC7721 cells,the relative VEGFA mRNA level was 1.56±0.92 (*P* < 0.05, n = 3). (**c**) VEGFA protein expression levels were up-regulated in pcDNA3.1-KLF8-transfected SMMC7721 cells, KLF8-overexpressing HCC cells (0.96 ± 0.09 *vs* 0.52 ± 0.05, P < 0.05, n = 3) had higher levels of VEGFA (0.86 ± 0.08 *vs* 0.38 ± 0.03, P < 0.05, n = 3) protein expression.

To further investigate the effects of KLF8 on VEGFA expression, pcDNA3.1-KLF8 was constructed to up-regulate KLF8 expression, and pcDNA3.1-KLF8 or pcDNA3.1 was transfected into SMMC7721 cells. KLF8 mRNA levels were higher in pcDNA3.1-KLF8-transfected HCC cells than in pcDNA3.1-transfected HCC cells; the relative KLF8 mRNA level was 472.66 ± 8.65 (*P* < 0.001, n = 3) (Fig. 2a). VEGFA mRNA levels were also up-regulated, and the relative VEGFA mRNA level was 1.56 ± 0.92 (*P* < 0.05, n = 3) (Fig. 2b).The protein levels of VEGFA and KLF8 were determined by western blotting; KLF8-overexpressing HCC cells (0.96 ± 0.09 *vs* 0.52 ± 0.05, P < 0.05, n = 3) had higher levels of VEGFA (0.86 ± 0.08 *vs* 0.38 ± 0.03, P < 0.05, n = 3) protein expression (Fig. 2c), which indicated that KLF8 up-regulation increases VEGFA mRNA and protein levels in HCC.

### KLF8 up-regulation induces VEGFA promoter activity

**Figure 3:**
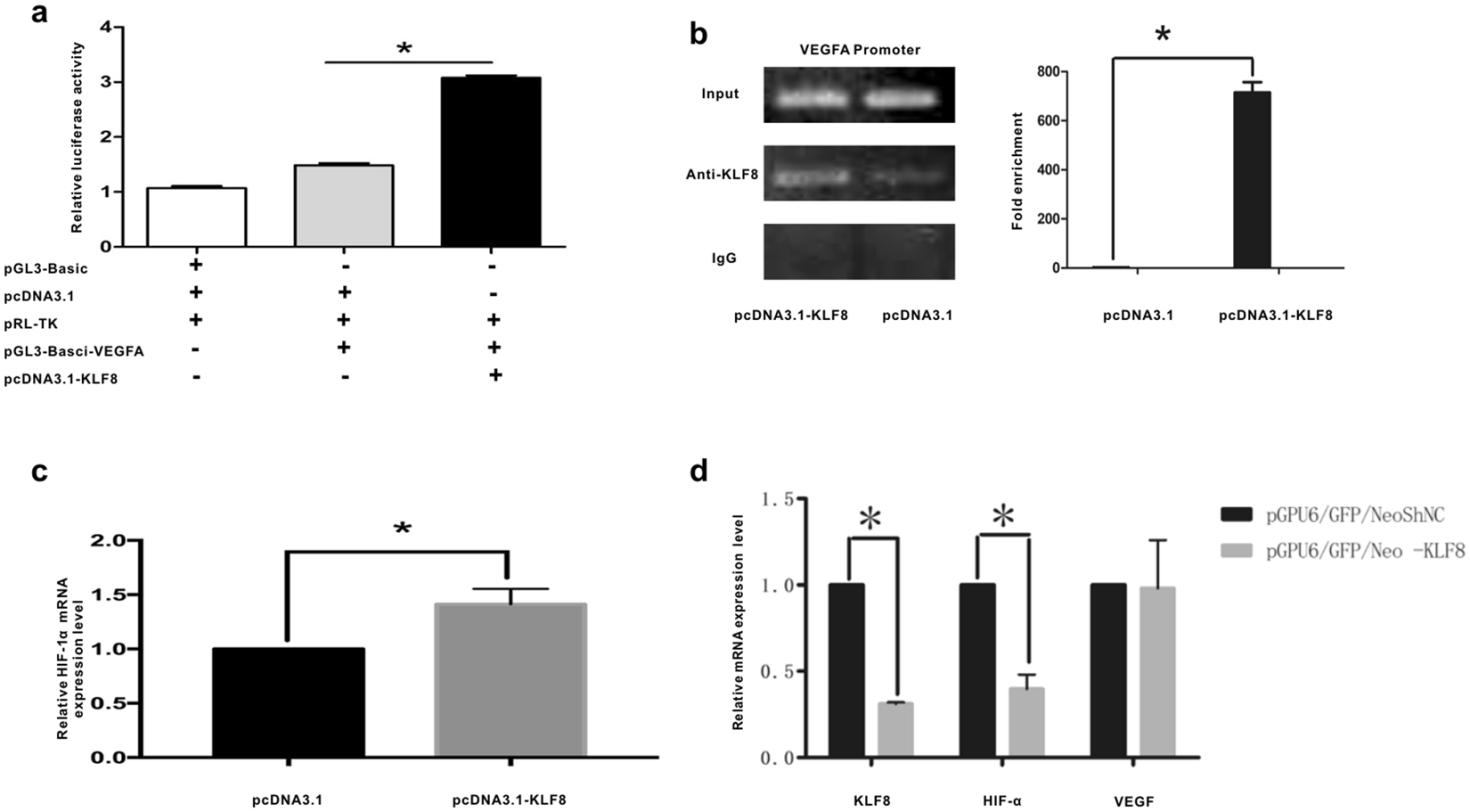
KLF8 induces VEGFA reporter activity by binding to the CACCC region of the VEGFA promoter and regulating HIF-1α expression. (**a**) The promoter region of VEGF-A (−2068/50 bp) was cloned from human genomic DNA, and the fragment was inserted into pGL3-Basic to construct pGL3-Basic-VEGFA-P plasmid. Dual-Luciferase Reporter Assay System was used to detect luciferase activity, the relative luciferase activity in pcDNA3.1-KLF8 group and pcDNA3.1 group was 3.08 ± 0.04 and 1.49 ± 0.04, respectively. (P < 0.0001, n = 3),which indicated that KLF8 induced VEGFA reporter activity. (**b**) Three sets of primers were used to amplify three “CACCC” sites of the VEGFA promoter region. ChIP assay real-time PCR results indicated that KLF8 binds to the “CACCC” site 637 nucleotides upstream of the VEGFA promoter region. Normal rabbit IgG was used as a negative control, pcDNA3.1-transfected SMMC7721 cells were used as a control group. The amplification for anti-KLF8 in KLF8-overexpressing SMMC7721 cells and pcDNA3.1 transfected SMMC7721 cells is 715.0 ± 42.23 *and* 2.15 ± 0.16 respectively. (p < 0.05, n = 3),the results indicated that KLF8 bound to the CACCC region of the VEGFA promoter. (**c**) Compared with pcDNA3.1-transfected SMMC7721 cells, the mRNA levels of HIF1-α were increased in pcDNA3.1-KLF8-transfected SMMC7721 cells (P < 0.05, n = 3). (**d**) Compared with that of SMMC7721 cells transfected with pGPU6/GFP/Neo-ShNC, the mRNA level of HIF1-α was decreased in SMMC7721 cells transfected with pGPU6/GFP/Neo-KLF8 (P < 0.05, n = 3), and the VEGFA mRNA levels were not different (p > 0.05).

Because KLF8 is a transcription factor, it is possible that KLF8 may regulate VEGFA expression at the transcription level. Therefore, we assessed whether KLF8 proteins could bind directly to the VEGFA promoter. The promoter region of VEGFA (−2068/50 bp) was cloned from human genomic DNA, and this fragment was inserted into pGL3-Basic to construct the pGL3-Basic-VEGFA-P plasmid. Promoter reporter assays were used to detect the effects of KLF8 up-regulation on VEGFA promoter activity. VEGFA promoter activity was induced significantly by KLF8 up-regulation (1.49 ± 0.04 vs. 3.08 ± 0.04, P < 0.0001, n = 3) (Fig. 3a).

### KLF8 binds to the CACCC region of the VEGFA promoter

KLF8 can function as either a transcription repressor or activator by binding to the GT-box (CACCC) promoter sequence via its three C-terminal C2H2 zinc fingers that are highly conserved among KLFs^9,14–17^. To confirm whether KLF8 binds to the CACCC region of the VEGFA promoter or not, ChIP assays were performed to identify the KLF8-binding region of the VEGFA promoter. Several primers were designed to amplify the CACCC sites of the VEGFA promoter. The amplification for anti-KLF8 in KLF8-overexpressing SMMC7721 cells and pcDNA3.1 transfected SMMC7721 cells is 715.0 ± 42.23 *vs*. 2.15 ± 0.16 (p < 0.05, n = 3) (Fig. 3b).This result indicated that KLF8 could bind to the CACCC region of the VEGFA promoter.

### KLF8 regulates the mRNA expression levels of HIF1-α

We also determined whether KLF8 regulated HIF1-α expression in HCC. pGPU6/GFP/Neo-KLF8 was constructed to down-regulate KLF8 expression, and pGPU6/GFP/Neo-ShNC was constructed as a control. Compared with that in pcDNA3.1-transfected SMMC7721 cells, the mRNA level of HIF1-α was increased (P < 0.05, n = 3) in pcDNA3.1-KLF8-transfected SMMC7721 cells (Fig. 3c). Moreover, compared with that of SMMC7721 cells transfected with pGPU6/GFP/Neo-ShNC, the mRNA level of HIF1-α was decreased (P < 0.05, n = 3) in SMMC7721 cells transfected with pGPU6/GFP/Neo-KLF8 (Fig. 3d). These data indicated that KLF8 up-regulation in HCC increases HIF1-α expression levels and that KLF8 down-regulation inhibits HIF1-α expression. However, VEGFA mRNA levels were not different in the HIF-1α-silenced group (p > 0.05). This finding indicated that other mechanisms may be involved in regulating VEGFA expression when KLF8 is down-regulated in HCC.

### KLF8 up-regulation regulates the expression of proteins in the PI3K/AKT signal pathway

**Figure 4:**
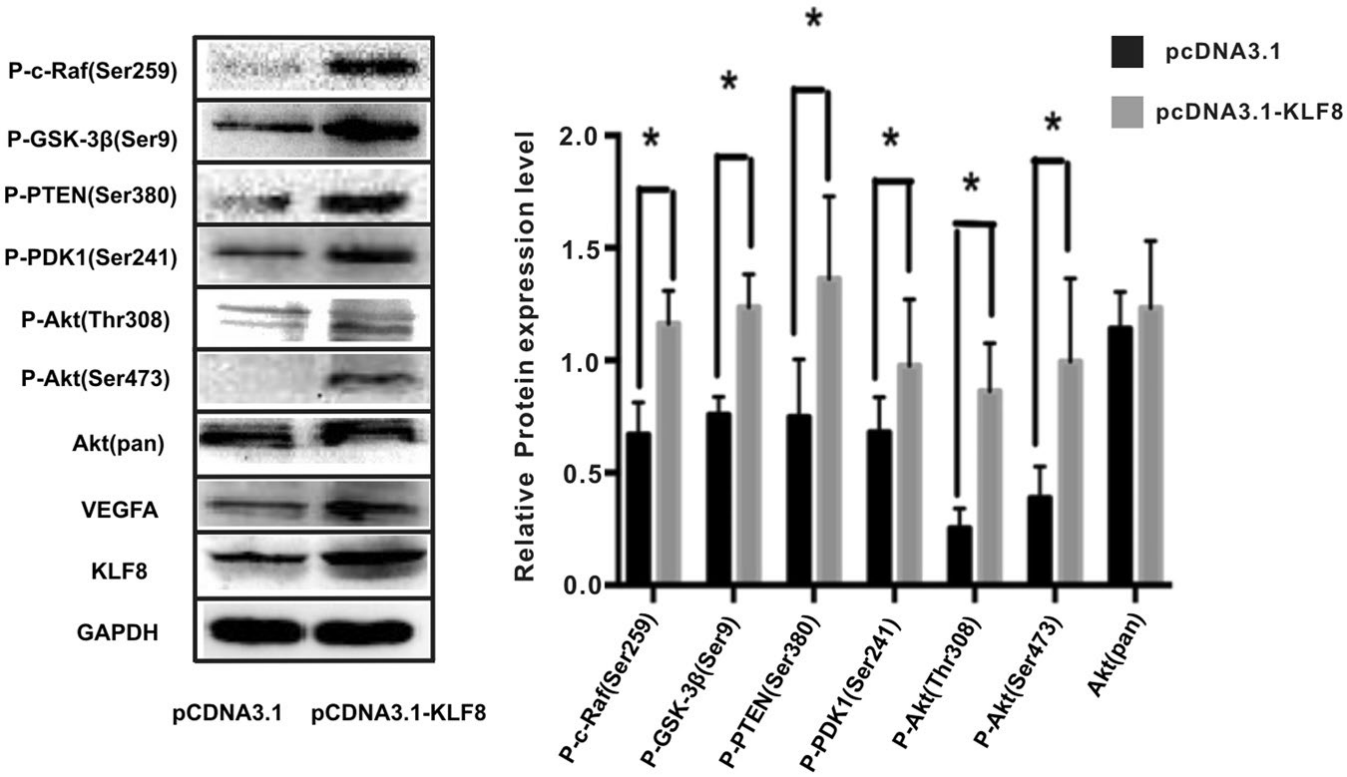
The expression of PI3K/AKT signaling proteins is increased in KLF8-overexpressing SMMC7721 cells. The expression levels of proteins in the PI3K/AKT signaling pathway in pcDNA3.1-KLF8-transfected SMMC7721 cells and pcDNA3.1-transfected SMMC7721 cells were detected by western blotting. The expression levels of P-c-Raf(Ser259), P-GSK-3β(Ser9), P-PTEN(Ser380), P-PDK1(Ser241), P-AKT(Thr308) and P-AKT(Ser473) were higher in SMMC7721 cells transfected with pcDNA3.1-KLF8 than in SMMC7721 cells transfected with pcDNA3.1 (P < 0.05, n = 3), and the AKT(pan) protein levels did not change significantly (p > 0.05, n = 3). The blots were from one gel and were then probed with different antibodies one at a time.

The PI3K/AKT signaling pathway is important in angiogenesis; to investigate the role of the PI3K/AKT signaling pathway in KLF8-mediated VEGFA up-regulation, the KLF8 expression plasmid pCDNA3.1-KLF8 was transfected into the SMMC7721 HCC cell line to up-regulate KLF8 expression, and the pCDNA3.1 plasmid was transfected as a control. The protein expression levels of KLF8, VEGFA and the PI3K/AKT signaling pathway proteins P-c-Raf(Ser259), P-GSK-3β(Ser9), P-PTEN(Ser380), P-PDK1(Ser241), P-AKT(Thr308), P-AKT(Ser473), and AKT(pan) were detected by western blotting. KLF8-overexpressing HCC cells had higher levels of VEGFA, P-c-Raf(Ser259) (1.16 ± 0.15 vs 0.67 ± 0.14), P-GSK-3β(Ser9) (1.24 ± 0.15 vs 0.76 ± 0.08), P-PTEN(Ser380) (1.36 ± 0.37 vs 0.75 ± 0.26), P-PDK1(Ser241) (0.98 ± 0.29 vs 0.68 ± 0.16), P-AKT(Thr308) (0.86 ± 0.21 vs 0.25 ± 0.09), and P-AKT(Ser473) (0.99 ± 0.37 vs 0.39 ± 0.14) (P < 0.05, n = 3), but the protein expression levels of AKT(pan) were not different (1.23 ± 0.29 vs 1.14 ± 0.16, P > 0.05, n = 3) (Fig. 4). These results indicated that the PI3K/AKT signaling pathway may participate in KLF8-mediated VEGFA expression changes. To verify the mechanism underlying KLF8-mediated PI3K/AKT signaling activation, we also measured focal adhesion kinase (FAK) levels. In KLF8-overexpressing SMMC7721 cells, FAK protein levels were increased significantly (0.56 ± 0.033 vs 0.82 ± 0.05, p < 0.05 n = 3) (Supplementary Figure 1a). We used FAKsiRNA to down-regulate FAK in SMMC7721 cells, the protein expression level of FAK decreased significantly in FAKsiRNA transfected SMMC7721(0.72 ± 0.07vs 0.34 ± 0.05, p < 0.05 n = 3), and the protein expression level of p-AKT was also down-regulated(0.52 ± 0.04 vs 0.22 ± 0.03,p < 0.05 n = 3). (Supplementary Figure 1b)

### Inhibition of the PI3K/AKT signaling pathway by LY294002 inhibits KLF8-induced VEGFA expression changes

**Figure 5:**
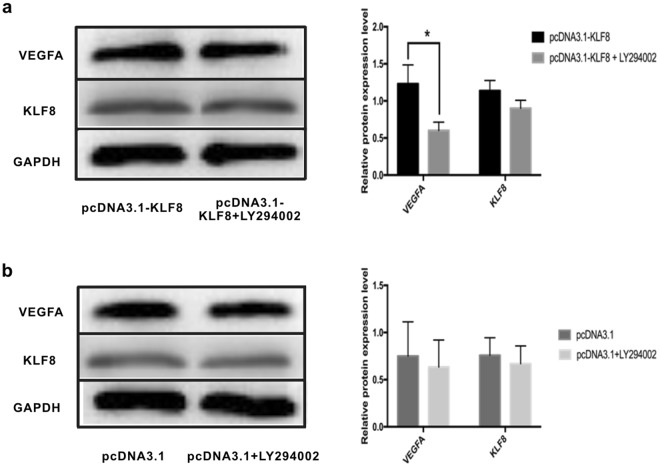
The PI3K/AKT signal inhibitor LY294002 inhibits KLF8-induced VEGFA protein expression. (**a**) VEGFA protein expression levels were significantly lower in LY294002-treated KLF8-overexpressing SMMC7721 cells than in DMSO-treated KLF8-overexpressing SMMC7721 cells (P < 0.05, n = 3). The KLF8 protein expression levels were not different. (**b**) In pcDNA3.1-transfected SMMC7721 cells, VEGFA expression levels did not change significantly after treatment with LY294002 (P > 0.05, n = 3). KLF8 protein expression levels were also unchanged.

We then determined the effect of PI3K/AKT inhibition on KLF8-mediated VEGFA expression changes. The HCC cell line SMMC7721 was transfected with pcDNA3.1 or pcDNA3.1-KLF8, and the PI3K/AKT inhibitor LY294002 was then added; DMSO was added as a control. In pcDNA3.1-KLF8-transfected HCC cells, VEGFA protein levels decreased significantly with LY294002 treatment compared to those of DMSO-treated HCC cells (0.60 ± 0.11 *vs* 1.23 ± 0.25, P < 0.05, n = 3), and KLF8 protein levels were not significantly different (0.90 ± 0.11 *vs* 1.14 ± 0.14, P > 0.05, n = 3) (Fig. 5a). Regarding the VEGFA protein levels in HCC cells transfected with pcDNA3.1, there were no differences between the LY294002-treated group and DMSO-treated group (0.63 ± 0.29 *vs* 0.75 ± 0.36, P > 0.05, n = 3); there were also no differences in the KLF8 protein levels (0.67 ± 0.19 *vs* 0.76 ± 0.19, P > 0.05, n = 3) (Fig. 5b).

**Figure 6:**
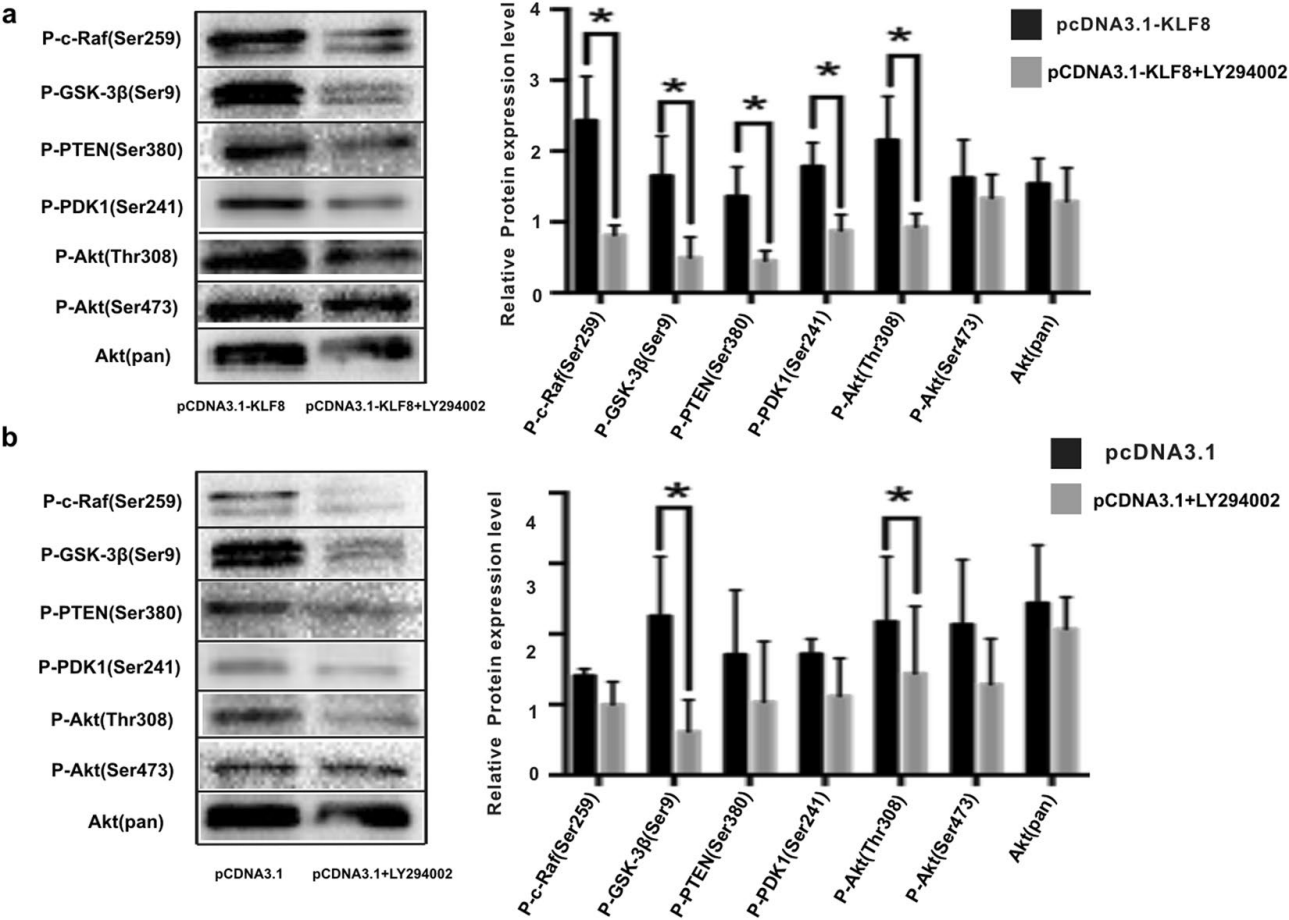
The protein expression levels of PI3K/AKT signaling proteins in pcDNA3.1-KLF8-transfected SMMC7721 cells and pcDNA3.1-transfected SMMC7721 cells after treatment with LY294002 or DMSO. (**a**) The protein expression levels of P-c-Raf(Ser259), P-GSK-3β(Ser9), P-PTEN(Ser380), P-PDK1(Ser241) and P-AKT(Thr308) were significantly lower in LY294002-treated KLF8-overexpressing SMMC7721 cells than in DMSO-treated KLF8-overexpressing SMMC7721 cells (P < 0.05, n = 3), and the protein expression levels of P-AKT(Ser473) and AKT(pan) were not different (p > 0.05). (**b**) In pcDNA3.1-transfected SMMC7721 cells treated with LY294002 or DMSO, the protein expression levels of P-c-Raf(Ser259), P-PTEN(Ser380), P-PDK1(Ser241), P-AKT(Ser473) and AKT(pan) were not different (p > 0.05), and protein expression levels of P-GSK-3β(Ser9) and P-AKT(Thr308) were decreased significantly after treatment with LY294002 (p < 0.05, n = 3). The blots were from one gel and were probed with different antibodies one at a time.

We also determined the expression levels of proteins in the PI3K/AKT signaling pathway. The protein expression levels of P-c-Raf(Ser259) (0.81 ± 0.14 *vs* 2.43 ± 0.62), P-GSK-3β(Ser9) (0.49 ± 0.29 *vs* 1.65 ± 0.56), P-PTEN(Ser380) (0.45 ± 0.14 *vs* 1.36 ± 0.42), P-PDK1(Ser241) (0.87 ± 0.23 *vs* 1.78 ± 0.34) and P-AKT(Thr308) (0.93 ± 0.19 *vs* 2.15 ± 0.62) were significantly lower in LY294002-treated KLF8-overexpressing HCC cells than in DMSO-treated KLF8-overexpressing HCC cells (P < 0.05, n = 3), and the protein expression levels of P-AKT(Ser473) (1.34 ± 0.33 *vs* 1.62 ± 0.54) and AKT(pan) (1.29 ± 0.47 *vs* 1.54 ± 0.35) were not significantly different (p > 0.05, n = 3) (Fig. 6a).

In pcDNA3.1-transfected HCC cells, the protein expression levels of P-c-Raf(Ser259) (0.70 ± 0.05 *vs* 0.49 ± 0.16), P-PTEN(Ser380) (0.85 ± 0.46 *vs* 0.52 ± 0.43), P-PDK1(Ser241) (0.86 ± 0.11 *vs* 0.56 ± 0.27), P-AKT(Ser473) (1.07 ± 0.46 *vs* 0.64 ± 0.32) and AKT(pan) (1.22 ± 0.41 *vs* 1.03 ± 0.22) were not different between the LY294002 and DMSO treatment groups (p > 0.05, n = 3), and the protein expression levels of P-GSK-3β(Ser9) (1.13 ± 0.42 *vs* 0.31 ± 0.23) and P-AKT(Thr308) (1.09 ± 0.46 *vs* 0.72 ± 0.48) were decreased significantly by LY294002 treatment (p < 0.05, n = 3) (Fig. 6b). These results suggested that the up-regulation of VEGFA by KLF8 might be inhibited by PI3K/AKT signaling pathway inhibitors. P-c-Raf, P-PTEN, P-PDK1, P-GSK-3β(Ser9), P-AKT(Thr308), and P-AKT(Ser473) may be involved in this process.

### KLF8-overexpressing HCC cells have a higher potential for inducing angiogenesis *in vitro* and *in vivo*

**Figure 7:**
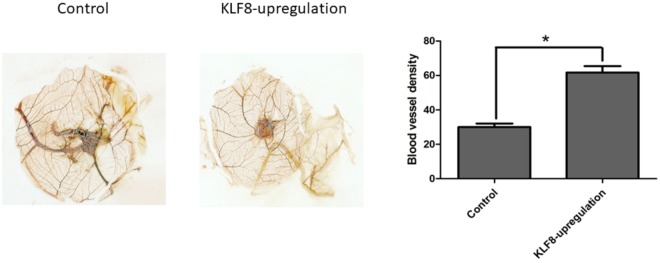
KLF8-overexpressing HCC cells have a higher potential for inducing angiogenesis. SMMC7721 cells transfected with pcDNA3.1 or pcDNA3.1-KLF8 were implanted in chicken embryos, and the angiogenesis induced by SMMC7721 cells was detected in this chicken chorioallantoic membrane (CAM) model. Statistical analyses indicated that CAMs implanted with KLF8-overexpressing HCC cells generated many more blood vessels than CAMs implanted with control HCC cells (61.67 ± 6.51 *vs* 30.00 ± 3.61, P < 0.01, N = 3).

**Figure 8:**
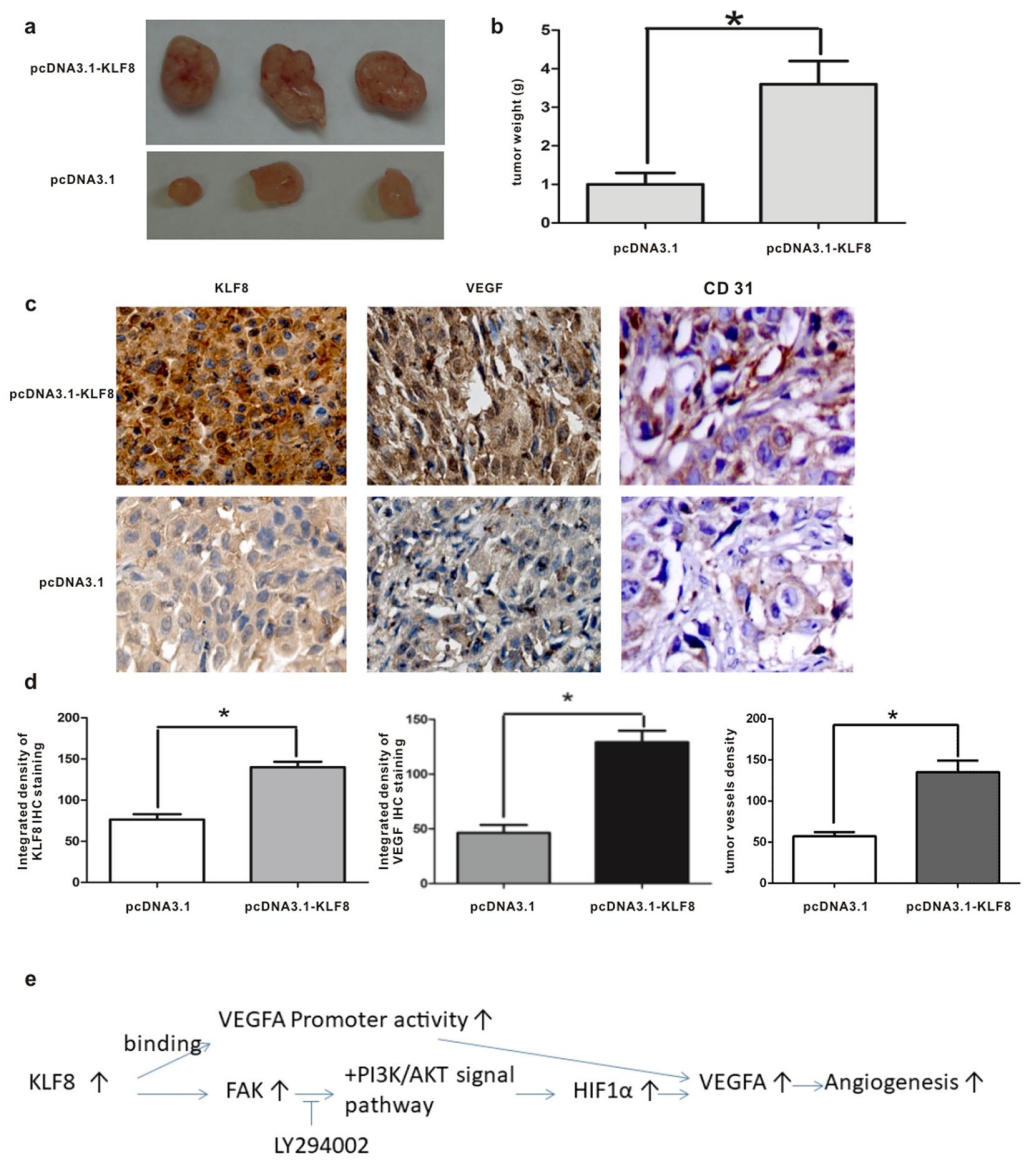
KLF8 promotes tumor growth and angiogenesis *in vivo* SMMC7721 cells (5 × 10^6^) transfected with pcDNA3.1-KLF8 or pcDNA3.1 were inoculated into the liver parenchyma of nude mice under ketamine/xylazine anesthesia after the abdomen was opened. All mice were monitored once every 3 days and sacrificed 5 weeks later. Tumor tissue sections were prepared, and immunoreactivity was analyzed as above using KLF8, VEGF and CD31 antibodies. (**a**,**b**) SMMC7721 cells transfected with pcDNA3.1-KLF8 had greater growth potential than SMMC7721 cells transfected with pcDNA3.1. In the nude mouse livers, the tumor weights were significantly higher in the SMMC7721-pcDNA3.1-KLF8 group than in the SMMC7721-pcDNA3.1 group (3.6 ± 0.6 g vs 1.0 ± 0.3 g, *P* < 0.01, n = 3). (**c**,**d**) VEGF and CD31 expression levels were detected by immunohistochemistry. The integrated density of VEGF staining was higher in the SMMC7721-pcDNA3.1-KLF8 group than in the SMMC7721-pcDNA3.1 group (129.2 ± 1.6 vs 46.3 ± 7.2, *P* < 0.01), and the tumor vessel density was significantly increased in the SMMC7721-pcDNA3.1-KLF8 group (135.2 ± 14.1 vs 57.3 ± 4.7, *P* < 0.01, n = 3) (**e**). Summary of proposed signaling pathways involved in the up-regulation of VEGFA by KLF8.

To determine the potential of KLF8-overexpressing HCC cells for inducing angiogenesis, SMMC7721 cells transfected with pcDNA3.1 or pcDNA3.1-KLF8 were implanted in chicken embryos, and the angiogenesis induced was then detected in this chicken chorioallantoic membrane (CAM) model. The tumor-centered blood vessel number (BVN) was counted, and the BVNs of the KLF8 overexpression group and control group were 61.67±6.51 and 30.00±3.61, respectively. KLF8-overexpressing SMMC7721 cells had a higher potential for inducing angiogenesis than control SMMC7721 cells (*P* < 0.01, n = 3) (Fig. 7). SMMC7721 cells transfected with pcDNA3.1-KLF8 had a higher growth potential than SMMC7721 cells transfected with pcDNA3.1. In nude mice livers, the tumor weights of the SMMC7721-pcDNA3.1-KLF8 group were significantly greater than those of the SMMC7721-pcDNA3.1 group (3.6 ± 0.6 g vs 1.0 ± 0.3 g, *P < *0.01, n = 3) (Fig. 8a,b). VEGF and CD31 expression levels were also detected by immunohistochemistry. The integrated density of VEGF staining was higher in the SMMC7721-pcDNA3.1-KLF8 group than in the SMMC7721-pcDNA3.1 group (129.2 ± 1.6 *vs* 46.3 ± 7.2, *P < *0.01, n = 6), and the tumor vessel density was significantly increased in the SMMC7721-pcDNA3.1-KLF8 group (135.2 ± 14.1 vs 57.3 ± 4.7, *P* < 0.01, n = 6) (Fig. 8c,d).

## Discussion

The growth and metastasis of cancer depend on angiogenesis. Vascular endothelial growth factor (VEGF) has been identified as a key mediator of tumor angiogenesis. The VEGF family includes VEGF-A, VEGF-B, VEGF-C, VEGF-D, VEGF-E, and placental growth factor (PlGF), and VEGF-A appears to be the most important in the growth of blood vessels in a variety of normal and pathological circumstances^18^. The effects of VEGF are mediated by endothelial cells via its receptors VEGFR-1 (Flt-1) and VEGFR-2 (KDR)^19^. Some tumor cells may also express VEGF receptors, and VEGF may act as an autocrine growth factor that stimulates the proliferation of some cancer cells^20^. In addition to the HIF1-α pathway, HBx protein activation, another mechanism that activates oncogenes; tumor suppressor gene loss or inactivation; and multiple signal transduction pathways, including Egr-1 and Sp1, may be involved in VEGF regulation in HCC. However, the mechanism of VEGF expression and its regulation in HCC are mostly unknown.

KLF8 is a member of the Krüppel-like C2H2 zinc-finger transcription factor family of proteins^9^. In our previous research, KLF8 up-regulation promoted HCC cell proliferation and invasion and inhibited apoptosis, and the over-expression of KLF8 increased HCC progression and metastasis. In the present study, we examined the expression of and relationship between KLF8 and VEGF in the tumor tissues of HCC patients. We found that the expression levels of KLF8 and VEGFA were highly related in HCC samples. KLF8-overexpressing HCC cells had higher VEGFA mRNA and protein levels. KLF8-overexpressing HCC cells had a higher potential for inducing angiogenesis according to chick chorioallantoic membrane (CAM) assays and a nude mouse HCC model.

Because KLF8 is a transcriptional factor, KLF8 up-regulation induced VEGFA promoter activity by binding to the CACCC region of the VEGFA promoter. PI3K/AKT signaling plays an important role in angiogenesis;^21^ once PI3K signaling is activated by PIP3, the pleckstrin homology (PH) domain of PDK1 is recruited to the plasma membrane, which results in the activation of membrane-associated AKT at threonine 308. AKT phosphorylation at serine 473 occurs independently via mammalian target of rapamycin complex 2 or is induced by PIP3. In addition, PIP3 binding activates PDK1 by promoting serine 241 autophosphorylation^22^. The mutation of PDK1 at serine 241 significantly reduces PDK1 activity toward AKT^23,24^. Activation of the EGFR/PI3K/AKT/mTOR pathway could increase VEGF expression by up-regulating HIF-1α^25^. Here, we showed that KLF8 up-regulation in HCC cells increased HIF1-α expression levels and that KLF8 down-regulation decreased HIF1-α expression levels. The induction of VEGF expression via KLF8 overexpression was blocked by the PI3K/AKT-specific inhibitor LY294002; in addition, the PI3K/AKT signaling pathway proteins P-PDK1(Ser241) and P-AKT(Thr308) decreased significantly, but the protein expression levels of P-AKT(Ser473) were not different. In pcDNA3.1-transfected SMMC7721 cells treated with LY294002 or DMSO, the protein levels of P-AKT (Thr308) were not different, and KLF8-overexpressing HCC cells had higher levels of P-PDK1(Ser241), P-AKT(Thr308) and P-AKT(Ser473). These results indicated that KLF8 up-regulation may act through the PI3K/AKT signaling pathway to increase P-PDK1(Ser241) levels; then, increased P-AKT(Thr308) or P-AKT(Ser473) protein levels could induce VEGFA protein expression.

Focal adhesion kinase (FAK) is a cytoplasmic protein tyrosine kinase that participates in regulating diverse cellular functions, such as cell spreading, migration, proliferation, and apoptosis^14^ .The FAK/PI3K/AKT signaling pathway plays an important role in HCC invasion^26^, and KLF8 overexpression causes the CXCL12/CXCR4-dependent activation of FAK^27^. Here, we showed that KLF8-overexpressing HCC cells had higher FAK levels (Supplementary Figure 1a), and the protein expression level of p-AKT decreased significantly in FAK down-regulated SMMC7721 cells(Supplementary Figure 1b),so it is possible that KLF8 activates PI3K/AKT signaling through FAK.

PTEN (phosphate and tensin homologue deleted on chromosome TEN) acts as a key negative regulator of the ligand-activated PI3K-AKT pathway;^28,29^ PTEN dephosphorylates phosphatidylinositol (3,4,5) triphosphate to its diphosphate (4,5) form, thus reducing the activation of AKT^30^. PTEN also has a restrictive role in angiogenesis^31^. The activation of Wnt signaling up-regulates VEGF expression^32^. GSK-3β is a negative regulator of Wnt signaling, and inhibiting GSK-3β increases VEGF promoter activity^33^. GSK-3β down-regulates HIF-1 and VEGF expression, thus inhibiting tumor angiogenesis *in vivo*^34^. Raf isoforms (ARAF, BRAF and CRAF in humans) initiate Raf/MEK/ERK signaling and can activate serine/threonine kinases; inhibiting the phosphorylation of c-Raf decreases the levels of p-MEK and p-ERK^35^. PI3K/AKT and Raf/MEK/ERK signaling cascades concurrently participate in angiogenesis via HIF-1α-mediated VEGF expression that is stimulated by notoginsenoside Ft1 (Ft1)^36^. In our study, KLF8-overexpressing SMMC7721 cells had higher levels of p-PTEN, P-GSK-3β and P-c-Raf, and these proteins levels decreased after LY294002 treatment. In pcDNA3.1-transfected SMMC7721 cells treated with LY294002 or DMSO, the protein levels of p-PTEN and P-c-Raf were not different, and P-GSK-3β protein expression levels decreased after LY294002 treatment. The roles of p-PTEN, P-GSK-3β and P-GSK-3β in KLF8-regulated angiogenesis in HCC need further investigation.

Taken together, our research showed that KLF8 induced angiogenesis in HCC by binding to the CACCC region of the VEGFA promoter to induce VEGFA promoter activity and through FAK to activate the PI3K/AKT signaling pathway to increase P-PDK1(Ser241) levels; then, increased P-AKT(Thr308) or P-AKT(Ser473) and HIF-1α levels induced VEGFA protein expression

### Experimental Procedures

#### Cell culture and tissue samples

The human hepatocellular carcinoma cell line SMMC7721 was grown in PRMI 1640 supplemented with 10% fetal bovine serum (FBS), 100 U/ml penicillin and 100 μg/ml streptomycin at 37 °C in a humidified incubator with 5% CO2. Fifty fresh tumor samples were collected randomly from HCC patients who underwent curative resection at the First Affiliated Hospital of Chongqing Medical University and Zhongshan Hospital of Fudan University China. All tissues were collected immediately upon tumor resection in the operating room, transported in liquid nitrogen, and then stored at −80 °C until use. These tissue samples were used for quantitative real-time polymerase chain reaction (qRT-PCR) assays. Paraffin-embedded HCC specimens were obtained from the Department of Pathology. HCC paraffin samples from adjacent sections were stained for KLF8 and VEGFA. Our study protocol complied with the Guidelines for the Performance of Research Involving Human Subjects established by the National Institutes of Health and the Committee on Human Research at Chongqing Medical University. The study was approved by the Committee on Human Research of Chongqing Medical University, and written informed consent was obtained from all patients.

### Total RNA Extraction and Reverse Transcription and Polymerase Chain Reactions

Total cellular and HCC tissue RNA was extracted using Trizol reagent (Invitrogen). cDNA was synthesized using a PrimeScript RT reagent kit (Takara, Dalian, China) according to the manufacturer’s protocol. PCR was performed with an initial denaturation at 95 °C for 30 s, followed by 40 cycles of denaturation at 95 °C for 1 min and annealing at 60 °C for 34 s in an ABI 7500 real-time PCR system. The primers used were as follows: KLF8: F: 5′-GCTCACCGCAGAATCCATACA-3′,

R: 5′-GTGCACCGAAAAGGCTTGAT-3′; GAPDH: F: 5′-TGGTATCGTGGAAGGACTCA-3′, R: 5′-CCAGTAGAGGCAGGGATGAT-3′; VEGF: F: 5′-ACTTTCTGCTGTCTTGGGTG-3′, R: 5′-CTGCATGGTGATGTTGGACT-3′; and HIF1-α: F: 5′-ATTACCCACCGC TGAAACGC-3′, R: 5′-TGAACTTTGTCTAGTGCTTCCATCG-3′. The relative expression levels were calculated using the 2^−△△Ct^ method.

### Cell transfection

Cells were seeded at a density of 5 × 10^5^ cells/well in 6-well plates one day before transfection. Four KLF8 shRNA expression plasmids pGPU6/GFP/Neo-KLF8 were constructed by Genepharma, Shanghai, and pGPU6/GFP/Neo-ShNC was used as a control; the most effective pGPU6/GFP/Neo-KLF8 was screened by real-time PCR and used to down-regulate KLF8 expression. FAK siRNA(sense: GUAUUGGACCUGCGAGGGA, anti-sense: UCCCUCGCAGGUCCAAUAC) was used to down-regulate FAK expression,

siRNA (sense:TTCTCCGAACGTGTCACGT, anti-sense: ACGTGACACGTTCGGAGAA) was used as control (GenePharma, Shanghai).The shRNA sequences were as follows:

KLF8-shRNA-1: GAAGACCTAGCATGCTACAAGCTCCAATTCAAGAGATTGGAGCTTGTAGCATGCTAGTTTTTG

KLF8-shRNA -2: GATCCAAAAACTAGCATGCTACAAGCTCCAATCTCTTGAATTGGAGCTTGTAGCATGCTAG.

pcDNA3.1-KLF8, pcDNA3.1, pGPU6/GFP/Neo-KLF8, pGPU6/GFP/Neo-ShNC plasmids and siRNAs were transfected by using Lipofectamine according to the manufacturer’s protocol (Invitrogen). RNAs were extracted 48 h after transfection for RT-PCR analyses. Total protein was extracted for western blot analyses.

### Construction of the VEGFA promoter-luciferase plasmids and co-transfection and luciferase assays

To assess the activity of the VEGFA promoter induced by KLF8, we constructed the pGL3-Basic-VEGFA-P plasmid. The promoter region of VEGF-A (−2068/50 bp) was cloned from human genomic DNA, and the fragment was inserted into pGL3-Basic. The constructs were verified by restriction endonuclease digestion and sequencing. SMMC7721 cells were seeded in 24-well plates at 0.5 × 10^5^ cells/well one day before transfection. SMMC7721 cells were co-transfected with 0.4 μg of the VEGF-A promoter luciferase reporter constructs, 0.2 μg of pGL3-basic-VEGFA promoter or pGL3-Basic reporter plasmids (Promega), and 0.2 μg of pcDNA3.1 (blank vector as a control) or pcDNA3.1-KLF8. Luciferase activity analyses were performed two days after transfection using a luciferase assay kit (Dual-Luciferase Reporter Assay System, Promega), and the data were normalized to Renilla luciferase activity.

The pRL-TK vector is a Renilla luciferase expression vector containing a thymidine kinase promoter. The working concentration of the pGL3-Basic-VEGFA, pGL3-Basic, pcDNA3.1-KLF8, and pcDNA3.1 plasmids was 100 ng/μL, and the working concentration of the pRL-TK Renilla plasmid was 10 ng/µL. In the experimental group, we used 2 µL of pGL3-Basic-VEGFA, 2 µL of pcDNA3.1-KLF8, 1 µL of pcDNA3.1, and 1.5 µL of pRL-TK. In the control group, 2 µL of pGL3-Basic, 1.5 µL of pRL-TK and 3 µL of pcDNA3.1 were used. To ensure the quality of the data, each transfection experiment was repeated at least three times.

### Chromatin Immunoprecipitation (ChIP) Assay

Nuclei for the ChIP assays were sonicated in shearing buffer, and the shearing effectiveness was confirmed by electrophoresis in ethidium bromide-stained agarose gels. The samples were then processed for immunoprecipitation using a kit (EZ-ChIP™ Chromatin Immunoprecipitation Kit, Millipore) and antibodies to KLF8 (Santa) according to the manufacturer’s instructions. After precipitation, the cross-linking was reversed, and PCR was carried out using 1 μL of each sample (input DNA dilution 1:10; immunoprecipitated fractions were undiluted) in PCR buffer (Qiagen, Valencia, CA) containing dNTPs (Invitrogen) and TAQ DNA polymerase (Qiagen) with the primers. Three sets of primers were used to amplify three “CACCC” sites of the VEGFA promoter region. ChIP assay real-time PCR results indicated that KLF8 binds to the “CACCC” site 637 nucleotides upstream of the VEGFA promoter region. Therefore, we used the 1386-5′GCTGTTTGGGAGGTCAGAAATAGG 3′-1409 and 1545-5′ ACGCTGCTCGCTCCATTCAC 3′-1526 primers; in addition, we used normal rabbit IgG as a negative control. pcDNA3.1-transfected SMMC7721 cells were used as a control group.

### Western blotting and immunohistochemistry staining

Total protein was prepared from the cell lines. Immunoblot experiments were performed according to standard procedures, and the following antibodies were used were used for the immunocytochemistry analysis: mouse anti-human monoclonal KLF8 (1:1000; Abnova); rabbit anti-human multiclonal P-c-Raf(Ser259), P-GSK-3β(Ser9), P-PTEN(Ser380), P-PDK1(Ser241), P-AKT(Thr308), P-AKT(Ser473), and AKT(pan) (Cell Signaling Technology); rabbit monoclonal anti-human VEGFA, mouse monoclonal anti-human GAPDH and mouse anti-human monoclonal KLF8 (1:1000; Abnova); anti-human focal adhesion kinase (FAK) (1:3000); and rabbit monoclonal anti-human VEGFA.

### Chick Chorioallantoic Membrane (CAM) and Nude Mice Tumor Growth Models

All animal procedures were conducted in accordance with international standards and were approved by the Commission for Ethical Experimentation on Animals of Chongqing Medical University.

Fresh fertile eggs were cleaned using a 1% solution of geramine and then incubated at 37.8 °C and 60%-80% relative humidity for 7 days. The shell was cut to create a small window (10 × 10 mm2), and the shell membrane was removed with sterile forceps to create an air chamber. The eggs were then sealed with sterile medical tape and incubated again. After 24 h, SMMC7721 cells were mixed with 100 µl of RPMI 1640 and 100 µl of Matrigel. When the mixture was nearly frozen, the cells were inoculated directly into the air chambers of the CAMs. The eggs were sealed with sterile medical tape and incubated at 37.8 °C and 60% humidity. After 120 h of incubation, the CAMs were fixed in methanol and acetone (1:1 volume) for 15 min. Then, they were cut and spread in distilled water. The status of the CAMs and the tumors (a 2-mm diameter was positive) could then be observed. The CAM vasculature was photographed using a scanner. The images were analyzed using an image analysis program, IPP, and the blood vessel density was calculated. A total of 5 × 10^6^ SMMC7721 cells transfected with pcDNA3.1-KLF8 or pcDNA3.1 were inoculated into the liver parenchyma of nude mice under ketamine/xylazine anesthesia after the abdomen was opened. All mice were monitored once every 3 days and sacrificed 5 weeks later. Tumor tissue sections were prepared, and immunoreactivity was analyzed as above using KLF8, VEGF and CD31 antibodies (BD PharMingen).

### Statistical Analysis

All data are shown as the means ± SD, and statistical analyses were performed with SPSS 15.0 for Windows (SPSS, Chicago, IL). Two-tailed Student’s *t-*tests were used for comparisons between groups. *P* values of < 0.05 were considered statistically significant.

## Electronic supplementary material

Expression of focal adhesion kinase (FAK) protein was increased in KLF8 up-regulated SMMC7721 cells, and FAK down-regulated SMMC7721 cells has lower p-AKT protein expression level.
