# Supplementary material for: Krüppel-like factor 8 regulates VEGFA expression and angiogenesis in hepatocellular carcinoma
Source: Sci Rep. 2018 Nov 27;8:17415. doi: 10.1038/s41598-018-35786-6 (PMC6258679; doi:10.1038/s41598-018-35786-6)

**Krüppel-like factor 8 regulates VEGFA expression and angiogenesis in hepatocellular carcinoma**

Sanuo Cheng^1,2*^, Xingping Zhang^1*^, Yali Xu^3^, Xiaobo Dai^1^, Jiachu Li^1^, Tao Zhang^1^, Xiaopin Chen^1^

^1^Department of Oncology, The First Affiliated Hospital of Chongqing Medical University, Chongqing, China

^2^Clinical Medical College, Chongqing Medical and Pharmaceutical College

^3^Department of Geriatrics, Chongqing General Hospital, Chongqing, China

*Sanuo Cheng and Xingping Zhang contributed equally to this work.

Correspondence to:

Jiachu Li,

Department of Oncology, The First Affiliated Hospital of Chongqing Medical University, Chongqing 400016, China

E-mail: li.jiachu@yahoo.com

Phone: +86-23-89011615

**Supplementary Figure 1: Expression of focal adhesion kinase (FAK) protein was increased in KLF8 up-regulated** **SMMC7721 cells ,and FAK down-regulated SMMC7721 cells has lower p-AKT protein expression level.**

a. SMMC7721 cells were transfected with pcDNA3.1-KLF8 to up-regulate KLF8 expression, and SMMC7721 cells transfected with pcDNA3.1 were used as a control. KLF8, FAK, and GAPDH protein expression levels were detected by western blotting. FAK protein levels were significantly higher in KLF8-overexpressing SMMC7721 cells than in the control cells (0.56±0.033 *vs* 0.82±0.05, p<0.05, n=3).

b. SMMC7721 cells were plated in a 6-well plate (6 × 10^5^ cells/well) and transfected with Control siRNA or FAK siRNA by using lipofectamine TM 2000, according to the manufacturer's instructions. Forty-eight hours after transfection, cells were collected for Western blotting to detect protein expression level of FAK, p-AKT. The protein expression level of FAK was down-regulated significantly in FAK siRNA transfected SMMC7721(0.72±0.07vs 0.34±0.05,p<0.05 n=3)，and the protein expression level of p-AKT was also down-regulated significantly(0.52±0.04 vs 0.22±0.03,p<0.05 n=3).


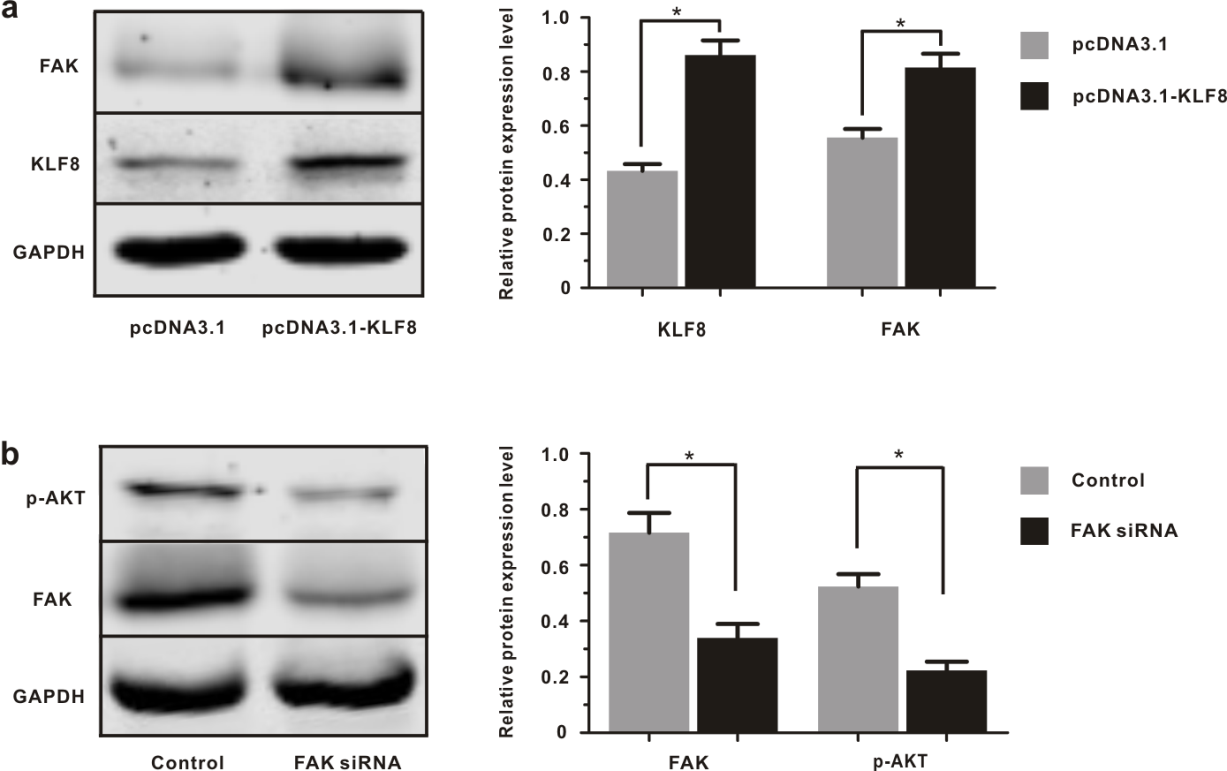

Supplement: Supplementary file 1 — Expression of focal adhesion kinase (FAK) protein was increased in KLF8 up-regulated SMMC7721 cells, and FAK down-regulated SMMC7721 cells has lower p-AKT protein expression level. [file 41598_2018_35786_MOESM1_ESM.docx]
